# Academic Leadership in the Time of COVID-19—Experiences and Perspectives

**DOI:** 10.3389/fpsyg.2021.648344

**Published:** 2021-04-20

**Authors:** Daniela Dumulescu, Alexandra Ileana Muţiu

**Affiliations:** ^1^Faculty of Psychology and Education Sciences, Babeş-Bolyai University, Cluj-Napoca, Romania; ^2^Faculty of Economics and Business Administration, Babeş-Bolyai University, Cluj-Napoca, Romania

**Keywords:** academic leadership, COVID-19 adaptability, distributed leadership, opportunities, qualitative research, thematic analysis

## Abstract

The COVID-19 pandemic has been a sharp reminder that large scale, unpredictable events always bring about profound changes with significant consequences on many levels. In light of lockdown measures taken in many countries across the world to control the spread of the virus, academics were “forced” to adapt and move to online settings all teaching, mentoring, research, and support activities. Academic leaders in higher education had to make decisions and to act quickly how were they to manage large educational communities, addressing students', teachers', and staff's needs, as well as society's needs. Using an inductive approach, our study seeks to highlight the main challenges faced by university leaders and to understand their responses to those challenges. The current study aims to investigate perceptions and experiences of academic leaders in a University in Romania during the COVID-19 pandemic. Our foci were the processes underlying their leadership decisions and actions during the second part of the 2019–2020 academic year. Data was collected through semi-structured interviews with 11 university and faculty leaders in Babeş-Bolyai University, Romania. The findings from the thematic analysis revealed three main themes emerging from participants' responses: “the leader's personal attributes,” “unity through decentralization,” and “opportunities to reinvent the university.” Good practices to manage academic institutions in times of crises and changes are discussed, along with insights into strategies for supporting higher education development during crisis and post-crisis times resulting into recommendations for increasing management effectiveness.

## Introduction

The COVID-19 pandemic brought several unpredictable challenges worldwide, forcing people to design and implement flexible solutions in order to adapt to the new reality. The crisis had a strong and deep impact on higher education at all levels. Due to the complexity of higher education institutions and their multi-faceted mission of teaching, conducting research, and contributing to society, managing change in academia during COVID-19 became a profound challenge for leaders.

Moving all the educational activities online showed that many universities already had all the tools and resources necessary for digitalization and for implementing effective decisions (Strielkowski, 2020). Academic leaders are increasingly tasked with making day-to-day critical decisions that will shape the future of their institutions. Additionally, in light of the urgent and dramatic shifts, and needs which arose in the last few months, universities have been confronted with various new issues and obligations toward students, staff, and academic audiences.

Thinking ahead, this pandemic period could be the restart button that higher education needs. It might be an opportunity for universities to recalibrate their organizations and to build a more efficient, accessible, and adapted set of offerings to the knowledge-based society in the post-pandemic world of work. Therefore, the main goal universities should assume is to be ready to increase their community impact in a competitive environment.

In the process of designing future universities, academic leaders will play an essential role. Given the increased complexity and diversity of situations that require immediate solutions, academic leaders will be making innovative decisions and responding to the needs (Al-Dabbagh, 2020). In the middle of such a acute crisis as the COVID-19 pandemic, the leaders act under high psychological pressure, with great expectations from members of various organizations for constant reassurance and support. Moreover, the pressure of time, ambiguity, the lack of information, and high level of stress, all increase the difficulty of the decision-making process. In this context, a wise leadership can help the organization become antifragile and resilient (Taleb, 2012), developing in the middle of a crisis through creative thinking, learning fast from experience of and adapting to the crisis, and through decentralized decision-making processes.

Making decisions in times of crisis requires great leadership competencies. Hence, analyzing the perceptions and experiences of academic leaders as decision-makers in a university may provide valuable insights about the decision-making process in complex educational institutions during major crises, such as the COVID-19 crisis.

### Academic Leadership

As a large institution, a university is governed by diverse structures and management bodies, from Rector, Vice-Rectors, and Deans to academic councils, department directors, administrative boards etc. Due to these particularities, academic leadership refers to different management roles and titles, varying from strategic management, administrative roles to transformational and visionary roles (Settles et al., 2019). Even in normal times, coordinating all those decision-makers for a common goal, can be very difficult. Many scholars investigating leadership in higher education proposed different models to conceptualize the dimensions of academic leadership. The model proposed by Ramsden (1998) describes the complexity and diversity of roles that leaders in higher education have. Moreover, that model shows the different levels of leadership in a university. According to Ramsden, there is a leadership dimension related to teaching, an effective leader should inspire his colleagues to feel excited about learning and to make good decisions about the educational process. Secondly, there is leadership related to the research dimension of a university, emphasizing the role of producing relevant knowledge assumed by higher education institutions. The next dimension in the model is related to strategy, vision, and networking, and focuses on setting a direction and advocating for it. Thus, an effective leader needs to formulate a clear vision for how to achieve that goal, which will provide a set of expectations as well as intrinsic motivation for colleagues (the latter being the main driving factor of academics). Additionally, this vision needs to be advocated eloquently to the rest of the university, in order to obtain the resources needed to implement it. At the same time, Ramsden focuses on another three aspects of leadership: the motivational dimension, recognition and interpersonal skills, and notes that a good leader needs to be able to lead both from the front (by example) as well as from the back (by recognition and support).

### Characteristics of Effective Academic Leadership in Crisis

According to the crisis management model proposed by Nathanial and Van der Heyden (2020) and applied to the COVID-19 crisis, there are some steps that every leader should be aware of in order to be effective in managing the crisis. In the initial phase, it is important to frame the crisis correctly and to communicate as early as possible the paths and methods that will be followed to manage the crisis. At the same time, the exploration of the problem together with different experts and formulating a clear strategy with well-defined indicators are important steps in managing the crisis. Next, the leader should communicate the decisions and the chosen scenarios, and then commit to action. Then comes the execution focus and constant monitoring of the actions. Finally, evaluating, learning, and adapting the efforts according to feedback is essential.

Beside the above-mentioned model of crisis management, some recent studies on leadership during the COVID-19 pandemic highlighted the most essential leader characteristics that bring effectiveness in time of crisis. Koehn (2020) proposed four key-competencies for an effective leader: providing meaningful roles, focusing on learning experiences, emotional agility, and acknowledging fear. Schwantes (2020) also mentioned four competencies needed to overcome the challenges associated with the COVID-19 crisis: flexibility, accounting for emotions, attention to other opinions, engagement. Dirani et al. (2020), emphasized some important roles and traits leaders should have in times of crisis: to be a sense maker, to be a technology enhancer, to have emotional stability, and to emphasize employee well-being and innovative communication in order to maintain the financial health of the organization.

Regarding academic leadership, the most important feature of leadership during the COVID-19 crisis that emerged in the latest publications on this topic brings distributed leadership to attention (Fernandez and Shaw, 2020). Usually, a university is a large institution with many faculties and departments, so a leader acting alone cannot succeed in a time of such a difficult crisis. The academic leader should be the one setting the strategy and the institutional priorities and at the same time giving their team the autonomy to assume the responsibility of their own decisions based on the specificity of their faculties/departments. Such a distributed leadership can be more effective in improving the quality and rapidity of decisions while increasing the sense of empowerment and motivation of each team (Kezar and Holcombe, 2017). The challenges of such a complex period require that top leaders engage in a explicit delegation of leadership that values the leadership potential of the people in their organization.

The academic decisions of shared leadership helped universities find solutions adapted to the crisis and to make local decisions benefiting from greater organizational agility, innovation, collaboration, and shared support (Fernandez and Shaw, 2020). A shared leadership paradigm helps a university to respond to a crisis through distributed leadership and an increase in responsibility at any organizational level (Kezar and Holcombe, 2017). Moreover, distributed leadership means making connections between people at all levels of the organization facing the challenges of a crisis and allows the transformation to be felt as meaningful for everyone. Moreover, it promotes psychological safety in the organization. So, the role of leaders is crucial in guiding the institution through finding the most appropriate solutions for empowering, developing a culture of trust, and orienting toward solutions that lead to effective results (Kezar et al., 2018).

At the same time, disrupting organizational norms, displaying courageous decisions, and engaging in proactive adaptation helped the transition from face-to-face activities to online, remote education. Academic leaders who see crises as strategic opportunities for innovation and using new technologies and techniques are the ones that bring the best results and practices. Effective leadership in crisis means risk taking, courage, flexibility, orientation toward goals and solutions, strategic vision and using an innovative approach meant to gain competitive advantage (Fernandez and Shaw, 2020). Leaders with great flexibility and adaptability and the capacity to perceive a crisis as an opportunity are effective in their decisions and have a strong capacity to navigate through uncertainty and to learn from experience (Ancona et al., 2007).

Due to the specificity of the COVID-19 crisis, which brings a lot of stress and uncertainty, the leader's personality characteristics and leadership style are also very important for building trust and accountability in the organization. From this point of view, a servant leadership emphasizing a collaborative, empathetic, emotionally stable leader personality can help build a strong community through commitment to the needs of the organization's members (Doraiswamy, 2012). A servant leader also focuses on the motivational and aspirational aspects and recognizes followers' need for psychological support and belonging (Eva et al., 2019), which suggests that if followers are treated as ends in themselves, rather than a means to an end, they will reach their potential and so perform optimally even in crisis (Waterman, 2011).

Organizational factors are equally important in facilitating effective solutions in response to crisis challenges. Making effective decisions in crisis means building organizational resilience. A resilient university is one that adapts and improves its responsiveness to challenges through absorbing adversities and going further (Dirani et al., 2020). The organizations that respond efficiently to crisis and changes are the ones that develop a culture of flexibility, learning from experiences, and orientation toward understanding the specificity of situations and of employees' issues (Caminiti, 2020). Moreover, an adaptative university is oriented, through accepting unpredictable contexts and finding ways to transform them into opportunities, toward sharing their own values and having an impact on their communities.

The characteristics of leaders and organizations we presented above, can offer a better picture for increasing the effectiveness of leadership in crisis, but they should be investigated in a more comprehensive framework. Due to the unpredictable and dynamic character of COVID-19 situation and our qualitative nature of the design, the meta-theoretical framework we adopted for our study is Complexity Leadership Theory (Uhl-Bien et al., 2007). This theory frames leadership as a complex, dynamic, and interactive relationship between leaders' organizational and situational factors, focusing on enabling adaptivity, learning and innovation in within a context of knowledge-based organizations (Uhl-Bien et al., 2007). Moreover, that theory emphasizes the idea that leadership strategies are embedded in context, with leaders valuing their characteristics to shape the dynamic emergent processes, in order to face the adaptive challenges requiring new patterns of decisions. More specific, the main leadership functions derived from those processes are adaptive, administrative, and enabling, interacting one to each other to every level.

### Rationale and Aim of the Study

There is a research gap regarding effective leadership of academic leaders in crises, especially in higher education. Recently, a few studies have been published investigating aspects of academic leadership in the COVID-19 pandemic. For example, Strielkowski and Wang (2020) investigated the way lockdown and restrictions have impacted higher education in the Czech Republic such that the forced digitalization resulted in a somewhat technological revolution. The paper also describes the implications of the COVID-19 pandemic for academic leadership, outlining predictions and provisions. In another study on academic leadership, Sá and Serpa (2020) have studied the opportunity provided by the current situation to reshape the higher education system in Portugal, discussing the role of leadership in digital development and the transformation of academic organizational culture. Suggestions for the reconceptualization of teaching methods, leadership models, communication channels and other useful insights and strategies are offered by the authors in a comprehensive manner that seeks to help tackle challenges and embrace opportunities.

Even though there are some valuable research contributions to the literature of academic leadership, the need to understand the characteristics and specificities of decisions made by leaders in higher education during the COVID-19 pandemic is important in order to understand and plan the effective development of universities for the future.

Therefore, we aim to provide a better understanding of academic leadership in crisis through a qualitative approach underlying one university's top academic leaders' experiences during COVID-19. We took a case-study approach and focused the investigation into one institution, Babeş-Bolyai University, in Romania. This was necessary to enable the analysis and to take into account the particular mission, values and specificity of academic environment and to make links between these and the leadership experiences and decisions. Moreover, the organizational case study approach brings value by undertaking an investigation into a phenomenon in its real context (Rowley, 2002).

Babeş-Bolyai University (UBB) in Cluj-Napoca is one of the oldest and largest universities in Romania (45.000 students). UBB occupies the highest position among the Romanian universities (in the National University Metaranking), being classified as an “advanced research and education university” by the Ministry of Education and as an “international university, with excellence in teaching and research” by The British QS STAR. UBB was also granted the “HR Excellence in Research” award in 2018 and has joined in 2020 the prestigious GUILD organization. One characterizing element of the university is the linguistic and cultural diversity, reflected in the carrying out and coordination of its educational activities, having three major lines of study (Romanian, Hungarian and German, plus other international languages in some of its schools), with 22 faculties offering 300 study programs in bachelor's, master's, and PhD degrees, as well as advanced postgraduate studies, from which students can freely choose. As a comprehensive university aiming at advanced research and education, UBB complies with the general mission of generating and transferring knowledge. Assuming these goals, the university is considering a strategic management plan and an efficient teaching plan, a balanced global development of the institution, adequate decision-making, ensuring democracy, collective participation, and transparency, and last but not least, assuming a set of quality principles that guide all activities within UBB. The university incorporates and advocates for the following values: tradition, excellence, freedom of expression, truth-seeking, integrity, equity, social responsibility, respect toward diversity, and intercultural cooperation. Regarding rectorship, the Hungarian and German lines are represented by one deputy Dean and one general deputy secretary, and in the Senate by one of its Vice-Presidents. The rectorship management office of the university is made of 1 Rector and 10 Vice-Rectors, working in unity to ensure the advancement and growth of the institution on several areas of development: administration, human resources, alumni, career and counseling, socio-cultural component, public relations and international relation, research, development and innovation, digitalization, patrimony, etc. All of these offices, together with the Deans of each faculty, the Council for Doctoral Studies Director, the representative of the General Administrative Directorate, and the students' prefect constitute Babeş-Bolyai University's Board of Directors.

Given the complexity of Babeş-Bolyai University, its academic leaders' reflections on the decision-making process and the results obtained during the COVID-19 crisis can bring insights into understanding leadership in higher education institutions.

More specifically, the objective of our research was to investigate perceptions and experiences of 11 academic leaders during the COVID-19 challenges, in order to understand the processes underlying leadership decisions during the second part of the 2019–2020 academic year.

Consequently, we aim to answer the following research questions: (1). What were the main characteristics of effective academic leadership during the COVID-19 challenges? (2). Which were the individual and organizational factors that facilitated leaders' decisions during the COVID-19 challenges?

## Materials and Methods

### Participants

The invitation to participate in this study was sent to all the Deans and Vice-rectors [*NB—this is the equivalent of Vice-President or Pro-Vice-Chancellors*] from Babeş-Bolyai University. The interviews were conducted with those who responded to that invitation in the time allotted to the study. Specifically, the data was collected from 11 academic leaders (5 Vice-Rectors and 6 Deans) from Babeş-Bolyai University, Romania. Two of them were women and nine men, ranging in age from 34 to 62 and having over 5 years of experience in academic leadership positions. All the participants were Associate Professors and Professors, having an academic background in the fields of: Biology, Business, Communication and Public Relations, Economics, History, Mathematics and Psychology. Their involvement in the study was voluntary, based on their available time and willingness to participate in the interview. They were informed about the interview's purpose, how it will be conducted, the estimated length of time it might take, and the confidentiality of the responses. Informant consent was obtained from every participant.

### Measures and Procedure

A semi-structured interview guide was developed with nine questions that explored the visions, strategies and actions taken through the lenses of personal and organizational effectiveness. The interviews were conducted in October and November 2020 and the participants were framed to respond by referring to their academic leadership experiences from March to October. Interviews lasted between 20 and 35 min and were conducted and moderated by the investigator. The discussions were audio recorded, transcribed verbatim and checked to ensure accuracy.

### Data Analysis

We authors analyzed the transcripts using the inductive thematic analysis based on the guidelines suggested by Braun and Clarke (2006), focused on the semantic and essentialist approach. Each of us conducted separately a systematic and independent analysis through the entire data. Both authors read the transcripts several times in order to generate the initial codes, then transformed them into potential themes. After checking the themes in relation with the codes, they were refined. Based on diagramming techniques, the themes and subthemes were clustered. In the final step, the most relevant excerpts from the interviews were read and checked again against the research questions and existing literature. To present the findings through excerpts and to maintain the confidentiality of the responses, we coded the participants with *Dean* and *Vice-Rector*, followed by a number from 1 to 5 for Vice-Rectors, and 1 to 6 for Deans.

## Results

The thematic analysis revealed three main themes that emerged from participants' responses: “the leader's personal attributes,” “unity through decentralization,” and “opportunities to reinvent the university.” In the section below, we present each of these themes, the subthemes we identified for each of the themes and example quotes from the interviews. We also discuss the theme and subthemes according to participants' academic background and managerial role (Vice-Rector and Dean).

### The Leader's Personal Attributes (Theme 1)

The first powerful theme that emerged from the participants' responses is related to essential personal characteristics of the leaders which underlined the leadership experiences during the first 6 months of the COVID-19 pandemic. The subthemes we extracted are responsibility, the power of experience and adaptability to changes. Those three personal attributes of leaders emerged as central for academic leadership in the perception of our participants.

Firstly, responsibility, seen as a catalyst for effective leadership, is expressed concisely and directly by these responses:

“*The ability to transform and acknowledge the responsibility you have*” *(Dean 3)*.

“*Our long-term vision is to empower every decision maker to do their job without passing it down to someone else*” *(Vice-Rector 3)*.

“*And considering the present circumstances [the current context] whoever is in a leading position nowadays is bound to take over some responsibilities without passing them down to the others, without delegating those partial decisions or responsibilities they are otherwise legally accountable for and should therefore, assume*” *(Dean 4)*.

“*And a change that brought, let's say, a little reluctance and maybe dissatisfaction among some colleagues was the idea of making everyone responsible to a higher degree*” *(Dean 5)*.

Secondly, previous leadership experience has been perceived as an effective factor in dealing with the pandemic:

“*I believe part of my professional experience in the field of communication has considerably helped uproot myself from this traditional sphere*” *(Dean 2)*.

“*Experience helped me in the first place*” *(Dean 3)*.

“*I think that this experience [i.e., as head of department] has greatly helped me in general: I would address certain issues no matter how complicated they might have seemed at the time. I have gained the necessary, even if insufficient, experience to start from somewhere and to face certain things*” *(Vice-Rector 5)*.

Thirdly, adaptability (an adaptable mindset) was also an important personal factor perceived as effective in dealing with various administrative, teaching, research, and communication situations:

“*So, whatever the challenges, we have the ability to adapt and UBB has the ability to adapt from this point of view, because this is what defines us*” *(Dean 4)*.

“*The flexibility it has shown. So, I think it was very important that in moments like these we did not proceed in a stubborn way… they expedited the development of the admission app and similar solution, which were hardly perfect, but, in the end, proved functional… we can't ask for perfection, especially when everything happens in the context of overnight crisis… Even then we still saw this flexibility, yes… people might have rejected these things. But we've accepted the challenge of exploring uncharted waters*” *(Dean 1)*.

“*Redrafting all the regulations pertaining to my portfolio, in light of the current situation, rethinking them so that we can include all the possibilities that may arise due to the pandemic*” *(Vice-Rector 1)*.

### Unity Through Decentralization (Theme 2)

The second theme which emerged from the responses is Unity through decentralization, revealing a community dimension of academic leadership, balancing autonomy with togetherness. The main subthemes refer to: setting the direction through guidelines, the autonomy of the faculties, and teams' power.

Firstly, the leadership process was characterized by the fact that the Rectorate set the direction through guidelines. This aspect was perceived by the respondents as a valuable asset of the university:

“*We all pull in the same direction, in other words, we try to manage this situation, we accept that this is what we have to deal with, and we are all part of the same team. I've never felt that each person wants something different. There was a shared goal, so to say*” *(Dean 3)*.

“*We've managed to tune our endeavors well, to act and speak as one: there were no absurd voices of dissent, arguing just for the sake of arguing against quite clear decisions that had to be taken drastically*” *(Dean 2)*.

“*There were some rules that still had to be followed. When a faculty had made a decision, it had to await the confirmation of the upper management before implementation*” *(Vice-Rector 1)*.

Secondly, the autonomy dimension was relevant for meeting the specific needs of the faculties:

“*Perhaps this was the wisest decision issued by the Rector's office: to give the faculties the choice of either going online or teaching face to face*” *(Dean 5)*.

“*The autonomy we had in deciding, as a faculty, whether to go online or face-to-face. In other words, respecting the singularity of each faculty guaranteed the consistency of a decision we had taken and complied with. And this was a positive thing because I felt that we could contribute to the welfare of the University*” *(Dean 1)*.

“*The fact that each faculty was allowed to choose a flexible or hybrid scenario for this period of teaching activities. And there was no imposition of a consensus: everyone in a certain way. That is, each faculty and each management team knows exactly which resources are available or how willing the teaching staff is to commit to face-to-face activities. The fact that we were allowed to put these things into perspective and make our own decision seemed extraordinary to me*” *(Dean 4)*.

“*The first and most important aspect is the mindset. The philosophy or the approach that the university board endorsed: to create general frameworks so that each faculty can make a specific decision that would facilitate a better control over and organization of activities. Should things go adrift, we try to put them back in place, but we always give the faculties enough wiggle room to act and think for themselves*” *(Dean 3)*.

Thirdly, the power of the teams was highlighted by all respondents, emphasizing the importance of trust in others' competence and collaboration.

“*Eventually, I hope my colleagues and I have managed to concur on encouraging each and everyone to bring something on the table*” *(Dean 2)*.

“*There are certain people I can collaborate with, because they have the proper approach. They also possess the right energy, the necessary strength and responsibility, that is clear. But for this, I wouldn't be successful…. I can be brilliant, but that is all. In the end, a collaborative environment is what matters*” *(Vice-Rector 5)*.

“*We shifted into high gear, we have been working under pressure and stress ever since March, but we have done this together, which alleviated some of the pressure we felt. The result was that we managed to streamline all legal regulations so that they could be applied by everyone*” *(Vice-Rector 3)*.

“*One aspect that helped me a lot was the top management's confidence that my team and I would have the ability to coordinate things well*” *(Dean 3)*.

“*I trust the people I coordinate, and I take the responsibility for each decision upon myself. This is very important*” *(Vice-Rector 4)*.

### Opportunities to Reinvent the University (Theme 3)

The third theme revealed more of a community dimension of leadership, focusing on the capacity of the university to become resilient and to grow in a post-pandemic world. Responses reveal two subthemes related to the future: the university's legacy for tomorrow and its openness to change.

Firstly, the university's legacy for tomorrow, as reflected by the actual practices implemented throughout the university for the pandemic situation, is perceived as being valuable for application even in non-pandemic conditions:

“*I believe that many of the things we have acquired now will be put into our everyday practice… all the networks we have created must, undoubtedly, be reinforced*” *(Dean 2)*.

“*I believe that some changes could not have been possible, were it not for the Covid situation. I look upon the present time as one that fosters change, and change is certainly a prerequisite for adaptation… somehow, people understood that they have to embrace change*” *(Vice-Rector 3)*.

Secondly, openness to change was perceived and experienced as an essential dimension of the institution facing the challenges:

“*I can say that the pandemic triggered latent ideas, some perhaps less visible to us, in the sense that until now, they did not rise as immediate opportunities: open online courses, workshops which are not only feasible but could also represent effective teaching sessions…and these have been long elicited, planned and needed, indeed*” *(Vice-Rector 5)*.

“*This (pandemic) is an opportunity to radically reconsider our view of the educational system…to be able to thoroughly redesign the vision of a Romanian faculty and to share this vision as an example of good practices*” *(Dean 3)*.

“*The present moment and context bring with them a unique opportunity to capitalize on our potential both in the way we understand our development and in the way we steer the progress of a faculty*” *(Dean 4)*.

## Discussions

The results of our study revealed novel insights about the personal and organizational factors contributing to the effectiveness of academic leaders' activity and decisions during the COVID-19 pandemic and supported the results existing in recent literature.

This research nonetheless probes more deeply into the processes of academic leadership. Analyzing the themes and subthemes which emerged in our research, we can identify processual connections between them. Firstly, there are the leader's personal attributes, emphasizing responsibility and adaptability, and building on previous experiences as a leader. Behind this combination of individual characteristics, we can infer there was a strong proactive attitude and an assumed risk-taking behavior, which helped the leaders find and create meaningful leadership experiences even in crisis (Fernandez and Shaw, 2020). Secondly, the leaders' personal attributes helped the university to adopt the strategy of *unity through decentralization*. Proving responsibility and adaptability, along with the experience, the university's top leaders adopted a strategy that allowed mid-level leaders (Deans) to express their leadership styles, offering them the freedom of action within a given general framework. Deans acted responsibly, identifying viable solutions to continue the activity during the pandemic period. Moreover, decentralization gave Deans the opportunity to create and apply new systems, to communicate in a new way, to find new advantages and solutions in order to be able to continue their activity. Once these new systems, procedures, and solutions were tested and applied, Deans understood their potential even in the post-pandemic period. Therefore, the themes represent parts of the evolution process of the university, but also reveal some differences among Deans and Vice-Rectors.

If we discuss the results of the Deans and Vice-Rectors and take their academic background into account, we can gain a more contextual insight. Vice-Rectors have insisted more on the attribute of responsibility. A possible explanation could be the fact that Vice-Rectors are more aware about the importance of the leadership experience and engaged in their work duties of setting the general framework for all the faculties. On the other hand, the Deans emphasized the power of personal adaptability, more than Vice-Rectors. Those differences can be explained through the fact that while the Vice-Rectors' tasks were to implement all the national laws and measures and to create an effective framework, whereas the Deans had to find ways to adapt to the specificity of their programs and faculties to that framework.

Another important aspect we observed in the responses is that responsibility is underlined by those respondents with background in Humanities and Social Sciences, more than those in Math and Science. The need of autonomy was also more strongly emphasized by the Deans, with richer descriptions, this being strongly related to their responsibility. On the other hand, the Vice-Rectors insisted more on the power of the teams, reflected in the Rectorate strategy of shared and distributed leadership. Regarding the theme *opportunity to reinvent the university*, there were no notable differences between Deans and Vice-Rectors, but it is worth mentioning that those with background in Business, Economics and Mathematics appeared more interested in this topic, with a specific preference for *openness to change*.

The findings are valuable if we consider that academic leaders are usually elected based on their academic and scientific prestige, not on their managerial background and education, which can be a barrier in the leadership roles (Elena-Pérez et al., 2011). For our participants, an important factor contributing to the internalization of their role, was their previous experience as academic leaders, which can be an important predictor of performance (Fiedler, 1994). Based on the previous discussion, distributed leadership played a crucial role in the university's policy, which enhanced trust and added relevant feedback loops.

Moreover, in allowing faculties to decide and act independently within a given framework, top leaders of the university showed trust in Deans and their actions (Montgomery, 2020). Empowered with trust, Deans conveyed it to their teams, as it appears from the excerpts presented. We notice here a two-way relationship between Rectorate and Deans and Deans and their teams. Thus, trust transcends the three themes: the self-perceived trust of leaders, manifested through responsibility and adaptability, is put into practice (capitalized at the institutional level) through the strategy of unity through decentralization of the university which goes even further, being reflected by the desire to reinvent the university. Consequently, in dealing with the pandemic crisis, the university acted “in the humanist spirit of trust and openness, to generate an organizational culture of solidarity and cooperation” (David, 2020, p. 5). Therefore, the university's values are assumed by its leaders and transmitted further to members. Sharing the same values amplifies collective efficiency and leads to unity in direction.

Trust has its own merits in developing efficient teams. The reverberation of trust facilitates a proactive attitude of the faculty members, which contributes to the increase of the teams' efficiency. This result is supported by the findings of Almutairi (2020) on effective leadership in higher education, which states that through their positive attitude to work, like encouraging innovation and not complaining about difficult tasks, managers increase the performance of faculty members.

The *feeling* of the team and the sense of community was perceived as being a powerful resource for implementing effective decisions and face the challenges, which emphasizes the important role of a distributed leadership and the value of controlling uncertainty through building meaningful actions for university members (Kohtamäki, 2019; Zafar et al., 2019). At the same time, sharing trust and common directions increased the psychological safety and effectiveness of members from the lower level of the organization (Samoilovich, 2020).

Thirdly, the individuality and the effectiveness of the teams was supported by organizational resilience and flexibility. The participants of our study perceived they were empowered by a general institutional orientation to learn, grow, and develop, despite the challenges. An organization that discovers its internal resources of adaptability and learning becomes more powerful in dealing with environmental demands (De Boer and Goedegebuure, 2009). The pandemic forced leaders to find new solutions, to adapt, and the results did not take long to appear: teaching platforms, new approaches for the teaching process, the advantages of offering education for students all over the world, new intra-university communication channels and so on. These achievements open new perspectives not only for the continued existence of universities, but also for their development.

The pandemic showed us new horizons and forced us to think from a new and innovative perspective. The challenge for academic leadership is how to encourage the necessary skillset and mindset shifts for all who work in higher education (Davis and Jones, 2014).

Furthermore, the current study's results confirm previous leadership theories and findings which conceptualize leadership as a complex process embedded in a social context. The academic leaders' perceptions revealed the interdependence of individual, organizational, and situational factors in supporting their decisions, implemented both on informal (emergent) as well as formal (hierarchical) processes (Bolden et al., 2012).

Even if the academic management roles define specific responsibility, the effective application of management tasks was strongly dependent on leadership self-efficacy (personal attribute), shared trust, common goals and perceiving the change and crisis as opportunity, which created engagement and coherence in the process.

These findings can be a starting point for investigating the complex dynamics of mechanisms underlying academic leadership in time of crisis (Uhl-Bien et al., 2007). Moreover, the themes we derived from the participants ‘responses, emphasized the importance of distributed leadership. As previous researchers have stated, distributing leadership can improve effectiveness in a crisis (Berjaoui and Karami-Akkary, 2020) and enable rapid responses and decision making (Kezar and Holcombe, 2017). If we investigate a deeper level, the relations between Deans, Vice-Rectors and Rector, corroborated with the trust of academic community, increased self-efficacy, facilitated an adaptive emotional climate and reduced the resistance to change, all of which seem to be essential for higher education leaders (Heffernan and Bosetti, 2020).

The value of seeing leadership as a dynamic and collaborative process, where the Rectorate articulate the strategic goals and the responsibility is distributed among Deans and heads of departments, as previous authors suggest (Ancona et al., 2007) was strengthened in our study. Although the results of the present research are concordant with leadership studies, they should be discussed in the context academic environment in Romania. Babeş-Bolyai University is a top university in Romania, it has a good internal and public brand, an effective collaboration with public administration and strong partnerships with key representatives of the social and business environment. Those factors contributed to a sense of empowerment and the trust the academic leaders perceived during COVID-19 crisis. Moreover, the effectiveness of one university's decisions “depends on relations of relative conflict or trust between the university and government and society” (Kai and Li, 2013, p.5) and our university has a great autonomy given by laws and policies, which gave leaders' the freedom to design and implement its own measures. Since “autonomy concerns the experience of acting with a sense of choice” (Hocine and Zhang, 2014, p. 140) and accountability for education and research's quality is correlated with autonomy, acting based on it, is relying on the university values expressed in its organizational culture. Babeş-Bolyai's core values are “tradition and excellence (through rationality and wisdom) in the modern humanistic spirit of trust and openness” (David, 2020, p.1). Being a university with a long tradition, and also a strong orientation toward future excellence, the experienced academic leaders perceived they were able to act accordingly. Last but not least, Babeş-Bolyai University is usually a key actor in providing good practices and models in higher education in Romania. From this point of view, our results can be a starting point for improving academic leadership in other universities.

## Conclusions

This case study and its analysis make a significant contribution to contemporary literature and research on academic leadership. To our knowledge and to date, it is the first study to empirically explore this topic in the context of the COVID-19 pandemic. With this aim to investigate the perceptions and experiences of Deans and Vice-Rectors regarding leadership processes during crisis, our research enriches the understanding over the leadership specificity in the most challenging times in the last few decades. The results discussed above should be seen in the light of the specificity of Babeş-Bolyai University. Academic leadership practices may vary across universities according to their differing organizational culture, tradition, mission etc. Also, our study's limitations are related to the reduced number of participants, their academic backgrounds, and the fact that the sample was based on voluntary participation. Therefore, future studies can address this issue by including more diverse and larger samples.

For a better understanding of the topic the perceptions of other stakeholders (head of departments, administrative staff, students, etc.) should be addressed. Despite these limitations, the findings provide insight into the important dimensions of academic leadership when faced with big environmental challenges. Hence, this paper provided several insights into the interaction and relationships between individual leaders (traits, attitudes, and background), organizational and contextual factors, thus offering a framework for investigating academic leadership from a comprehensive model.

Our findings will inform academic leadership strategies by providing insights into effective dynamic processes of leadership characterized by empowering every person in strengthening the relation between adaptive needs and administrative identity, as embedded in the context (Koehn, 2020). From a practical perspective, this study can contribute to raising awareness of the importance of leadership processes in difficult times and of the needs of leadership training initiatives to foster change, innovation, and adaptation for finding the best ways to address local, national, and global challenges.

The findings can also guide the development of leadership and management tools and recommendations for academics and provide insightful perspectives on the topic. The future is already here, and a new mindset, new attitudes and practices will have to be incorporated into the new reality of universities, effectively harnessing the experience gained during the COVID-19 time.

## Data Availability Statement

The raw data supporting the conclusions of this article will be made available by the authors, without undue reservation.

## Ethics Statement

The studies involving human participants were reviewed and approved by Babes Bolyai University Ethics committee. The patients/participants provided their written informed consent to participate in this study.

## Author Contributions

All authors listed have made a substantial, direct and intellectual contribution to the work, and approved it for publication.

## Conflict of Interest

The authors declare that the research was conducted in the absence of any commercial or financial relationships that could be construed as a potential conflict of interest.
